# A Participatory Model for Cocreating Accessible Rehabilitation Technology for Stroke Survivors: User-Centered Design Approach

**DOI:** 10.2196/57227

**Published:** 2024-08-23

**Authors:** Andrew Kerr, Madeleine Grealy, Milena Slachetka, Chioma Obinuchi Wodu, Gillian Sweeney, Fiona Boyd, David Colville, Philip Rowe

**Affiliations:** 1 Department of Biomedical Engineering, University of Strathclyde, Wolfson Centre, 106 Rottenrow, Glasgow, G4 0NW, United Kingdom, 44 01415482855; 2 Department of Psychological Sciences & Health, University of Strathclyde, Glasgow, United Kingdom

**Keywords:** rehabilitation, rehabilitative, rehab, rehabilitation technology, accessibility, accessible, stroke, design, participatory, participatory design, participatory designs, participatory model, participatory models, user-centred, user-centered, user-focused, digital health, digital technology, digital intervention, digital interventions, participatory medicine, technology

## Abstract

**Background:**

Globally, 1 in 3 people live with health conditions that could be improved with rehabilitation. Ideally, this is provided by trained professionals delivering evidence-based dose, intensity, and content of rehabilitation for optimal recovery. The widely acknowledged inability of global health care providers to deliver recommended levels of rehabilitation creates an opportunity for technological innovation. Design processes that lack close consideration of users’ needs and budgets, however, mean that many rehabilitation technologies are neither useful nor used. To address this problem, our multidisciplinary research group have established a cocreation center for rehabilitation technology that places the end user at the center of the innovation process.

**Objective:**

This study aims to present the participatory cocreation model that has been developed from our center and illustrate the approach with 2 cases studies.

**Methods:**

The model is built around user participation in an intensive rehabilitation program (2-hour sessions, 2‐5 times per week, and 8-week duration), supervised by qualified therapists but delivered exclusively through commercial and prototype technology. This provides participants (chronic stroke survivors with movement and/or speech disability) with a rich experience of rehabilitation technology, enabling them to provide truly informed feedback, as well as creating an observatory for the research team. This process is supported by short-term focus groups for specific product development and a longer-term advisory group to consider broader issues of adoption and translation into everyday health care.

**Results:**

Our model has been active for 3 years with 92 (92%) out of 100 participants completing the program. Five new technologies have evolved from the process with further ideas logged for future development. In addition, it has led to a set of cocreated protocols for technology-enriched rehabilitation, including recruitment, outcome measures, and intervention structure, which has allowed us to replicate this approach in an acute hospital ward.

**Conclusions:**

Suboptimal rehabilitation limits recovery from health conditions. Technology offers the potential support to increase access to recommended levels of rehabilitation but needs to be designed to suit end users and not just their impairment. Our cocreation model, built around participation in an intensive, technology-based program, has produced new accessible technology and demonstrated the feasibility of our overall approach to providing the rehabilitation that people need, for as long as needed.

## Introduction

Across the world, 1 in 3 people live with a health condition that could benefit from rehabilitation [1]. Delivering effective levels of rehabilitation to meet this global demand is beyond the reach of most, if not all, state-run health services, not least because of the inadequate workforce [2,3]. This means that most people will either receive suboptimal rehabilitation or no rehabilitation at all. Consequently, recovery from disabling conditions such as stroke is not simply a function of severity but will depend on an individual’s capacity to access additional rehabilitation. Technology has reached the point of maturity where could it help address this large unmet need in an equitable manner. Rehabilitation technology, such as virtual reality and robotics, has been shown to improve function across a range of conditions, for example, stroke [4] and Parkinson disease [5], as well as age-related disability [6]. Access to this technology, however, has been described as poor or nonexistent in the public sector of many countries, including the United Kingdom [7].

Besides the initial challenge of access, the subsequent abandonment of prescribed technology (rehabilitation and assistive) is common; for example, Sugawara et al [8] reported that more than 50% of upper-limb prostheses were not used after prescription. Many reasons are given for the nonuse of technology in rehabilitation. Sweeney et al [7] found reasons that stem from both therapists (eg, lack of training) and patients (eg, poor motivation). To overcome these barriers and increase the use of technology in rehabilitation, a number of recommendations have been proposed, including improved usability, clinical evidence of effectiveness, value for money, and conforming to self-management programs [9].

These recommendations require the involvement of end users throughout the design process, for the people in need of rehabilitation to be cocreators of the technology and be involved at different stages in the development process both as determiners and evaluators of these technologies.

Cocreation is a relatively new approach in health care. The idea originated in marketing and management [10], driven by the desire for bottom-up economics and greater personalization. The collaborative approach quickly spread into other domains including health care, where it has been used to develop services such as rehabilitation [11] and the design of assistive devices [12]. Irrespective of the field of study, cocreation is the practice of identifying and empowering relevant stakeholders (user groups) to collaborate in the process of finding solutions to a problem affecting the group. Its application in health care has been described using different terms such as co-design and coproduction [13]. The common idea behind cocreation is the involvement and partnership between the researcher or designer and the end users of the product, services, or intervention in generating concepts and evaluating products [14] .

While cocreation can address many of the user-based issues identified with rehabilitation technology (usability, access, and adherence) [11], a potential weakness is the imbalance between designers and users in their knowledge and experience. Such an imbalance may be reflected in the outcomes. Users’ knowledge of rehabilitation technology is likely limited in the range of technology and limited to their day-to-day experience of using them as part of a rehabilitation program. A participatory approach [15] would allow users to gain the necessary knowledge to make meaningful contributions to the design process.

In 2021, our research group set up a cocreation center for rehabilitation technology [16], aiming to develop accessible rehabilitation using a cocreation approach that is informed by users who have completed, or are completing, an 8-week, technology-based rehabilitation program [16,17]. This paper describes the formal and informal cocreation processes that developed from our center and presents 2 cases studies to demonstrate how specific devices have benefited from our participatory cocreation approach.

## Methods

### Participants

Details of our research center (participants, intervention, staff, and outcome measures) are provided in previous publications [16,17]. In the interests of clarity, they are briefly described here. Participants living with disabilities caused by stroke (mobility, communication, and cognition) were invited to attend an 8-week rehabilitation program at the University of Strathclyde. Participants were recruited through invitations distributed by a medical charity: Chest Heart and Stroke Scotland. Interested individuals attended an initial meeting to assess eligibility (more than a year since a stroke diagnosis; well enough, and able, to attend at least twice a week; and had a physical and/or communication or cognitive disability resulting from stroke), and their baseline measures were recorded.

### Ethical Considerations

The study was approved by the University of Strathclyde ethics board (UEC20/08) and all participants provided informed consent process. Participant data were anonymized, and there was no compensation for study participation.

### Intervention

A goal-setting interview and baseline measures of mobility, communication, and cognition helped our research therapists (physiotherapist and occupational therapist) to design an intensive, personalized rehabilitation program. The programs were delivered exclusively through technology (eg, treadmills, power-assisted exercise machines, tablet apps, virtual reality, upper-limb robots, balance-training systems, and functional electrical stimulation) but supervised in small (n=5‐10) circuit-based classes by at least 1 therapist. Each session was 2 hours long, for which participants can attend daily but must agree to attend at least 2 sessions a week for the 8-week period. We called the program Technology Enriched Rehabilitation Gym (TERG) to encapsulate training with technology designed to address the range of impairments resulting from stroke.

### Outcome Measures

Standard, validated measures of mobility (eg, Berg Balance Scale and the Ten Meter Walk Test) and global impact (Stroke Impact Scale) were recorded immediately before and after the program. These have been well described in our other publications, including pilot data on outcomes [17].

### Cocreation Activities

Our cocreation activities were aimed at either the development of specific devices or informing the strategy for implementing the TERG model into practice. For device development, short-term, purposively selected focus groups were formed from individuals (n=5-8) currently attending the TERG to provide focused user feedback on the device. The number of focus groups varied (typically 3‐5) and could have extended into future groups, in which case individuals were invited to continue contributing.

Translating and integrating our TERG model into everyday rehabilitation practice is the long-term aim of our center. To achieve this, we have formed a User Advisory Group that meets formally 3 times a year and provides feedback on specific plans and ideas around implementation in community and hospital settings.

**Figure 1.**
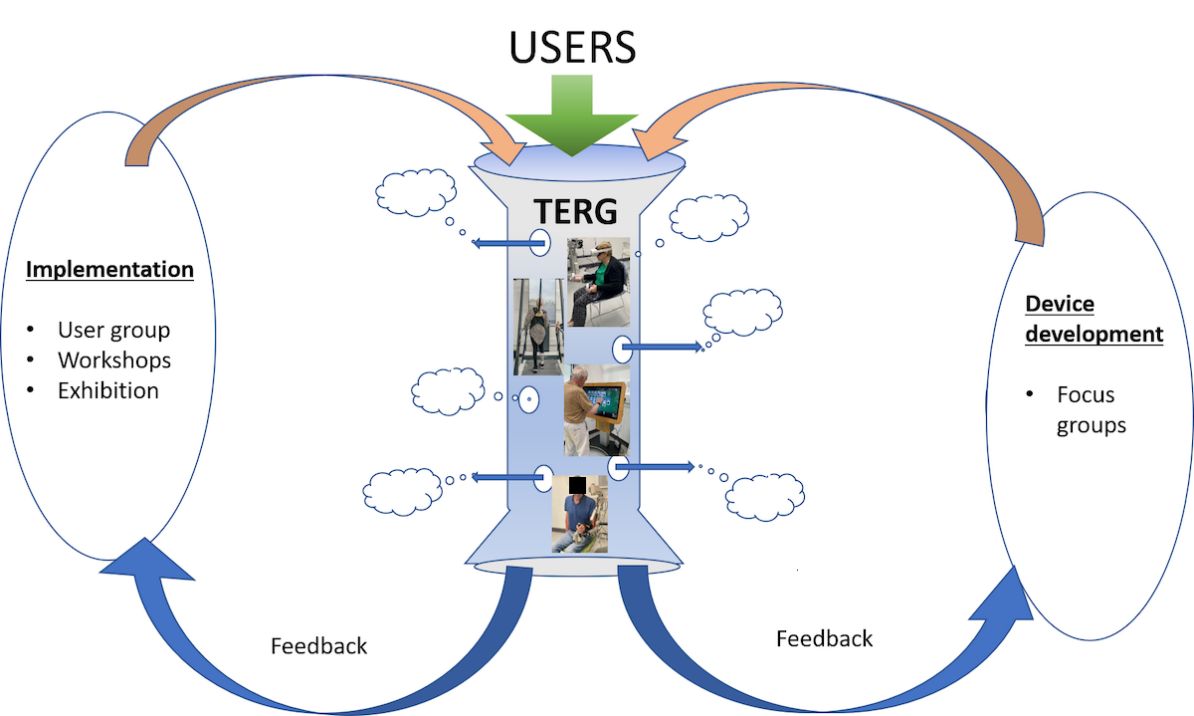
Overview of the participatory cocreation process showing the core 8-week program and related focus and user groups. TERG: Technology Enriched Rehabilitation Gym.

The activities described so far represent formal methods of cocreation. The opportunity to observe and work closely with these heterogeneous users as they carry out their technology-based rehabilitation provided our multidisciplinary research group (therapists, engineers, and scientists) with a rich dataset of daily informal observations on how users interact with technology and how this evolves over the course of 8 weeks as users learn and improve. These informal observations were documented in the laboratory book and reviewed by the team at the end of each group. This more informal mechanism has arguably provided a greater volume of feedback on devices and led to several new ideas that are currently being explored. A graphical overview of the whole cocreation model is presented in Figure 1.

To illustrate how the model functions practically, we present 2 case studies in the following section.

## Results

### Overview

Our participatory model of cocreation has been active for almost 3 years (from September 2021 until June 2024), with 92 (92%) out of 100 recruited participants fully completing the 8-week program. Feedback from these individuals has contributed to the design process of 5 rehabilitation technologies, with further concepts logged for future development. Case studies for 2 of these technologies are presented below. Critically, participant feedback, along with data on feasibility (safety and adherence) and impact on function, has also produced a set of cocreated protocols for technology-enriched rehabilitation, including recruitment, outcome measures, and intervention parameters. This has allowed us to replicate our approach in an acute hospital ward.

### Case Study 1: Design of a Low-Cost Hand Device for People With Hemiplegia

The aim was to design a technology that could improve the hand flexibility and function of people with moderate-to-high levels of spasticity that was accessible in community settings (low cost, easy to use, and did not require professional supervision) including low-income countries, was comfortable, and supported self-management.

The design process followed the UK Design Council’s Double Diamond model [18], which promotes divergent (creating a range of solutions) and convergent (narrowing solutions down through a set of criteria) thinking. The model supports a cocreation approach with users (in this case, rehabilitation professionals and stroke survivors) contributing to the discovery and delivery phases of this iterative design model through observations of technology interactions, focus groups, and interviews.

The design process started by observing stroke survivors participating in the TERG model and engaging them in discussions related to hand rehabilitation. This early discovery phase provided general design criteria (comfort and ease of use) and important features that were further refined by a focus group of rehabilitation engineers (n=8) to ensure feasibility in terms of manufacturing. Three potential designs were then presented to 2 user groups: (1) rehabilitation professionals (physiotherapists and occupational therapists; n=9) experienced in this area and (2) stroke survivors (n=6), to reduce this list to a single design that was the most appropriate to solving the problem.

A semistructured interview (choice of in person or virtual) was conducted by a researcher (COW) for each participant, during which 3D models of the 3 concepts were presented to generate opinions on key attributes (usability, comfort, and effectiveness). The interviews were recorded, transcribed, and anonymized. Thematic analysis was then used to identify common themes in the resulting data and used to reduce the list of devices to a single preferred device that would be built for further hands-on evaluation.

A prototype of the final choice has been tested for feasibility and acceptability by a new group of stroke survivor attending the center. The device is currently going through further refinement as part of a process to prepare it for commercialization.

### Case Study 2: Design of a Rehabilitation Dosage and Intensity Monitoring System

This case study aimed to develop a system for monitoring rehabilitation dosage and intensity to allow stroke survivors and clinicians to gauge activity against the *National Clinical Guidelines for Stroke* [19]. The system tracks and logs the dosage and intensity of rehabilitation activities users partake in throughout their time at the cocreation center for rehabilitation technology, thereby supporting users in their recovery process. Central to its foundation was a co-design process, meticulously planned over a year through 4 focus groups. This methodological approach ensured the inclusion of direct feedback from participants, fostering a rapport that enriched the design process with iterative refinements and consistent insights.

Analysis from these sessions revealed a notable gap in the transition from prescribed to self-managed rehabilitation, often leading to reduced engagement. Yet, it also highlighted a persistent motivation among individuals to pursue adequate rehabilitation, particularly when supported by peers. This insight steered the development toward leveraging peer support to bolster self-rehabilitation motivation. Consequently, the project led to the collaborative design of a system that should not only facilitate home- and community-based stroke rehabilitation but also improve the engagement and motivation of a person to complete their rehabilitation exercises.

Using these insights, the project embarked on the development of a mobile app with accompanying hardware to support home- and community-based stroke rehabilitation. This development process also used gamification principles to make said rehabilitation activities more engaging, with a strong emphasis on social involvement and accessible peer support. Further on in the design and development process, the involvement of stakeholders from the stroke community, participants of the cocreation model, health care professionals, and researchers ensured that the device not only met the unique needs of its users but also aligned with evidence-based rehabilitation principles.

## Discussion

### Principal Findings

We have described our participatory approach to the cocreation of rehabilitation technology and presented 2 case studies to illustrate the process and highlight the potential benefits of this approach. Our model expands the concept of cocreation beyond surveys, questionnaires, and interviews or focus groups [20]. Contributions from end users are enriched by their participation in an 8-week, technology-based rehabilitation program. Feedback is consequently highly informed, detailed, and authentic with the opportunity to compare technologies. This in-depth feedback is critical for designing technology that is fit for the “real world” [9].

The participatory nature of our model has also created an ideal observatory for engineers (biomedical and design) to collect data on the interactions between stroke survivors and rehabilitation technology. This has led to a number of new device concepts being drafted and adjustments to commercial technology, for example, alterations to hand grips and equipment portability, which have been accepted by our industrial partners. A surprising outcome from these observations and informal discussions with participants has been the desire to integrate technology, for example, balance and speech therapy training, and track these activities on a common platform. This is now a focused area of our activity.

Case study 1 demonstrates that our cocreation method can complement standard design models such as the UK Design Council’s Double Diamond model [21]. Similarly, case study 2 followed the Medical Research Council framework for the design of complex medical interventions and devices [22]. Our model ensures that the users’ voice strongly influences each phase of these innovation frameworks and guidelines and fulfills explicit requirements to engage stakeholders (Medical Research Council framework) and involve users [21].

### Limitations

In presenting this model, we recognize that there are some limitations. First, the volunteers attending our center may be more motivated and generally more positive toward rehabilitation than the average stroke survivor, since they have actively sought the opportunity for more rehabilitation. Their opinions may therefore be biased and not entirely generalizable. To address this potential bias, we have recently started a version of our center in a hospital setting where all eligible patients with stroke are offered the opportunity to experience technology-enriched rehabilitation.

The process may also raise issues around intellectual property, since a number of people contribute to technology development. This requires the involvement of an experienced research office and a legal framework that recognizes and protects different contributions.

Finally, we recognize that our model is not implementable in most engineering departments and industrial settings due to a lack of resource (equipment and therapy staff). This places greater importance on the need for collaboration across the rehabilitation engineering sector.

### Conclusion

There is an urgent need to develop rehabilitation technology that is fit for purpose and capable of supporting the recommended levels of rehabilitation. Our multidisciplinary group has developed a model of cocreation where stroke survivors with related disabilities participate in a technology-enriched rehabilitation program that captures meaningful feedback and contributions from end users on specific device development, including new concepts, as well as developing a model that can be widely adopted in everyday practice. We have presented this novel model for developing rehabilitation technology for discussion and included 2 illustrative case studies.

## Abbreviations

TERG: Technology Enriched Rehabilitation Gym
